# Bis{5-[(2-propyn-1-yl­oxy)meth­yl]-1,3-phenyl­ene}-32-crown-10

**DOI:** 10.1107/S1600536811046435

**Published:** 2011-11-09

**Authors:** Yu Yang, Zhi-kai Xu, Deng-ke Yang, Shao-wu Pan, La-sheng Jiang

**Affiliations:** a School of Chemistry and Environment, South China Normal University, Guangzhou 510006, People’s Republic of China

## Abstract

The mol­ecule of the title compound {systematic name: 17,35-bis­[(2-propyn-1-yl­oxy)meth­yl]-2,5,8,11,14,20,23,26,29,32-deca­oxatricyclo­[31.3.1.1^15,19^]octa­triaconta-1(37),15,17,19 (38),33,35-hex­a­­ene}, C_36_H_48_O_12_, has crystallographic inversion symmetry and adopts a chair-like conformation. The polyether bridges of the macrocycle adopt *gauche* conformations and the cavity of the macrocycle is collapsed. In the crystal structure, there are weak inter­molecular C—H⋯O hydrogen bonds driven in part by the elevated acidity of acetylenyl H atoms.

## Related literature

For applications of crown ethers, see: Gokel *et al.* (2004 ▶); Raymo *et al.* (1999 ▶) and of bis­phenyl­ene crown erthers, see: Loeb (2007 ▶); Fang *et al.* (2010 ▶); Kay *et al.* (2007 ▶). For cryptands, see: Zhang *et al.* (2010 ▶). For supra­molecular inter­locked structures, see: Xu *et al.* (2011 ▶) For the synthesis of bis­(5-hy­droxy­methyl-1,3-phenyl­ene)-32-crown-10, see: Gibson & Nagvekar (1997 ▶) and for the synthesis of the title compound, see: Xu *et al.* (2010 ▶). 
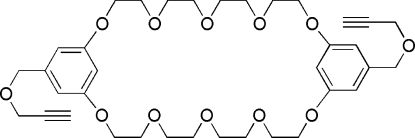


## Experimental

### 

#### Crystal data

C_36_H_48_O_12_
*M* *_r_* = 672.74
Triclinic, 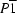

*a* = 9.2256 (13) Å
*b* = 9.8561 (14) Å
*c* = 10.0808 (14) Å
α = 97.213 (2)°
β = 98.658 (2)°
γ = 99.226 (2)°
*V* = 883.9 (2) Å^3^
*Z* = 1
Mo *K*α radiation
μ = 0.09 mm^−1^
*T* = 298 K
0.64 × 0.32 × 0.10 mm

#### Data collection

Bruker APEXII CCD diffractometer
Absorption correction: multi-scan (*SADABS*; Sheldrick, 1996 ▶) *T*_min_ = 0.965, *T*_max_ = 0.991
4551 measured reflections
3108 independent reflections
2350 reflections with *I* > 2σ(*I*)
*R*_int_ = 0.020

#### Refinement

*R*[*F* ^2^ > 2σ(*F* ^2^)] = 0.042
*wR*(*F* ^2^) = 0.113
*S* = 1.05
3108 reflections
217 parameters
H-atom parameters constrained
Δρ_max_ = 0.26 e Å^−3^
Δρ_min_ = −0.21 e Å^−3^

### 

Data collection: *APEX2* (Bruker, 2008 ▶); cell refinement: *SAINT* (Bruker, 2008 ▶); data reduction: *SAINT*; program(s) used to solve structure: *SHELXS97* (Sheldrick, 2008 ▶); program(s) used to refine structure: *SHELXL97* (Sheldrick, 2008 ▶); molecular graphics: *SHELXTL* (Sheldrick, 2008 ▶); software used to prepare material for publication: *SHELXTL*.

## Supplementary Material

Crystal structure: contains datablock(s) global, I. DOI: 10.1107/S1600536811046435/ld2029sup1.cif

Structure factors: contains datablock(s) I. DOI: 10.1107/S1600536811046435/ld2029Isup2.hkl

Supplementary material file. DOI: 10.1107/S1600536811046435/ld2029Isup3.cml

Additional supplementary materials:  crystallographic information; 3D view; checkCIF report

## Figures and Tables

**Table 1:** Hydrogen-bond geometry (Å, °)

*D*—H⋯*A*	*D*—H	H⋯*A*	*D*⋯*A*	*D*—H⋯*A*
C13—H13*B*⋯O6^i^	0.97	2.49	3.247 (2)	135
C18—H18⋯O4^ii^	0.93	2.54	3.203 (2)	128
C18—H18⋯O5^ii^	0.93	2.52	3.431 (3)	166

Symmetry codes: (i) 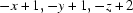; (ii) 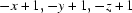.

**Fig. 1.**
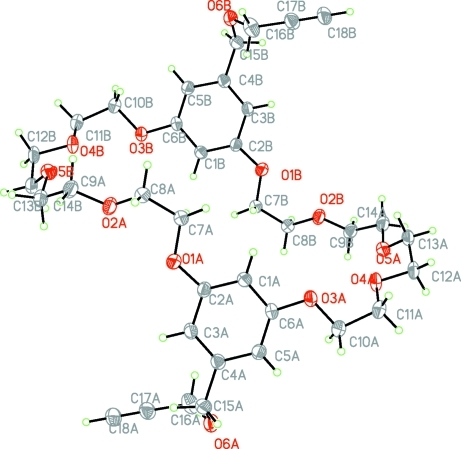
View of the title compound showing the atom-labelling scheme. Ellipsoids are drawn at the 50% probability level.

## supplementary crystallographic information

### Comment

Crown ethers are important building blocks in supramolecular chemistry and have been widely used in materials and biological sciences for sensors and switches (Gokel *et al.*, 2004; Raymo *et al.*, 1999). Recently, bisphenylene crown ethers, such as bisparaphenylene-34-crown-10 (BPP34C10) and bismetaphenylene-32-crown-10 (BMP32C10), attracted great interests and were extensively used for construction of interlocked molecules (Loeb, 2007), mechanically bonded macromolecules(Fang *et al.*, 2010) and molecular machines(Kay *et al.*, 2007). Their wide uses are mainly because bisphenylene crown erther hosts can form relatively stable molecular complexes with electron deficient paraquat derivatives by virtue of multiple noncovalent interactions, such as hydrogen bondging and charge-transfer interactions. As part of our project to explore novel crown ether-based cryptands (Zhang *et al.*, 2010,) in supramolecular self-assembly (Xu *et al.*, 2010) and interlocked structures(Xu *et al.*, 2011), we tackled the synthesis of bisacetylene-substituted BMP32C10, an important precursor to cryptands. We envisioned that the title compound could be obtained by the reaction of bis(5-hydroxymethyl-1,3-phenylene)-32-crown-10 with propargyl bromide in the presence of sodium hydride.

As shown in Fig. 1, the title compound has crystallographic inversion symmetry in the solid state. The phenyl rings are at a centroid-centroid distance of 9.422 Å and they are arranged in an edge-to-edge conformation rather than a face-to-face one. The polyether bridges of the macrocycle adopt a *gauche* conformation and the cavity of the macrocycle is collapsed. The molecule as a whole adopts a chair-like conformation. Weak intermolecular C—H···O hydrogen bonds driven by the elevated acidity of acetylene hydrogen were observed.

### Experimental

The title compound was synthesized from bis(5-hydroxymethyl-1,3-phenylene)-32-crown-10 (Gibson, *et al.*, 1997) which was reacted with sodium hydride and propargyl bromide (Xu *et al.*,2010). Colourless block crystal of the title compound suitable for X-ray diffraction analysis was obtained by slow evaporation of its acetone solution at room temperature.

### Refinement

The H atoms were positioned geometrically and allowed to ride on their parent atoms, with C—H = 0.95 - 0.99 Å, and *U*_iso_=1.2–1.5 *U*_eq_(C).

### Crystal data

**Table:**

C_36_H_48_O_12_	*V* = 883.9 (2) Å^3^
*M**_r_* = 672.74	*Z* = 1
Triclinic, *P*1	*F*(000) = 360
Hall symbol: -P 1	*D*_x_ = 1.264 Mg m^−^^3^
*a* = 9.2256 (13) Å	Mo *K*α radiation, λ = 0.71073 Å
*b* = 9.8561 (14) Å	µ = 0.09 mm^−^^1^
*c* = 10.0808 (14) Å	*T* = 298 K
α = 97.213 (2)°	Block, colourless
β = 98.658 (2)°	0.64 × 0.32 × 0.10 mm
γ = 99.226 (2)°	

### Data collection

**Table:**

Bruker APEXII CCD diffractometer	3108 independent reflections
Radiation source: fine-focus sealed tube	2350 reflections with *I* > 2σ(*I*)
graphite	*R*_int_ = 0.020
φ and ω scans	θ_max_ = 25.2°, θ_min_ = 2.1°
Absorption correction: multi-scan (*SADABS*; Sheldrick, 1996)	*h* = −11→10
*T*_min_ = 0.965, *T*_max_ = 0.991	*k* = −7→11
4551 measured reflections	*l* = −12→10

### Refinement

**Table:**

Refinement on *F*^2^	Primary atom site location: structure-invariant direct methods
Least-squares matrix: full	Secondary atom site location: difference Fourier map
*R*[*F*^2^ > 2σ(*F*^2^)] = 0.042	Hydrogen site location: inferred from neighbouring sites
*wR*(*F*^2^) = 0.113	H-atom parameters constrained
*S* = 1.05	*w* = 1/[σ^2^(*F*_o_^2^) + (0.0506*P*)^2^ + 0.1259*P*] where *P* = (*F*_o_^2^ + 2*F*_c_^2^)/3
3108 reflections	(Δ/σ)_max_ < 0.001
217 parameters	Δρ_max_ = 0.26 e Å^−^^3^
0 restraints	Δρ_min_ = −0.21 e Å^−^^3^

### Special details

**Table:**

Geometry. All e.s.d.'s (except the e.s.d. in the dihedral angle between two l.s. planes) are estimated using the full covariance matrix. The cell e.s.d.'s are taken into account individually in the estimation of e.s.d.'s in distances, angles and torsion angles; correlations between e.s.d.'s in cell parameters are only used when they are defined by crystal symmetry. An approximate (isotropic) treatment of cell e.s.d.'s is used for estimating e.s.d.'s involving l.s. planes.
Refinement. Refinement of *F*^2^ against ALL reflections. The weighted *R*-factor *wR* and goodness of fit *S* are based on *F*^2^, conventional *R*-factors *R* are based on *F*, with *F* set to zero for negative *F*^2^. The threshold expression of *F*^2^ > σ(*F*^2^) is used only for calculating *R*-factors(gt) *etc*. and is not relevant to the choice of reflections for refinement. *R*-factors based on *F*^2^ are statistically about twice as large as those based on *F*, and *R*- factors based on ALL data will be even larger.

### Fractional atomic coordinates and isotropic or equivalent isotropic displacement parameters (Å^2^)

**Table:**

	*x*	*y*	*z*	*U*_iso_*/*U*_eq_	
C1	0.19258 (19)	0.18786 (18)	0.55393 (17)	0.0405 (4)	
H1	0.0968	0.1897	0.5717	0.049*	
C2	0.21585 (18)	0.15530 (18)	0.42298 (16)	0.0393 (4)	
C3	0.35977 (19)	0.15509 (19)	0.39575 (17)	0.0428 (4)	
H3	0.3744	0.1335	0.3068	0.051*	
C5	0.45832 (19)	0.21882 (18)	0.63403 (17)	0.0427 (4)	
H5	0.5393	0.2404	0.7049	0.051*	
C6	0.31507 (19)	0.21810 (18)	0.65978 (16)	0.0392 (4)	
C8	−0.13388 (19)	0.0898 (2)	0.18269 (18)	0.0472 (5)	
H8A	−0.1242	−0.0054	0.1536	0.057*	
H8B	−0.2380	0.0914	0.1867	0.057*	
C7	−0.04096 (18)	0.1423 (2)	0.32005 (17)	0.0456 (4)	
H7A	−0.0407	0.2406	0.3455	0.055*	
H7B	−0.0801	0.0923	0.3878	0.055*	
C9	−0.1580 (2)	0.1224 (2)	−0.04624 (17)	0.0513 (5)	
H9A	−0.2653	0.1062	−0.0510	0.062*	
H9B	−0.1291	0.0348	−0.0762	0.062*	
C10	0.4014 (2)	0.2810 (2)	0.89901 (17)	0.0519 (5)	
H10A	0.4619	0.3688	0.8916	0.062*	
H10B	0.4639	0.2107	0.8987	0.062*	
C11	0.3400 (2)	0.2938 (2)	1.02859 (17)	0.0520 (5)	
H11A	0.2712	0.2091	1.0311	0.062*	
H11B	0.4206	0.3065	1.1054	0.062*	
C12	0.2334 (2)	0.4423 (2)	1.17067 (16)	0.0492 (5)	
H12A	0.3250	0.4829	1.2333	0.059*	
H12B	0.1886	0.3588	1.2018	0.059*	
C13	0.1296 (2)	0.5426 (2)	1.16786 (17)	0.0513 (5)	
H13A	0.0356	0.4998	1.1098	0.062*	
H13B	0.1102	0.5687	1.2587	0.062*	
C14	0.1140 (2)	0.7742 (2)	1.13577 (19)	0.0553 (5)	
H14A	0.1367	0.8184	1.2299	0.066*	
H14B	0.0076	0.7388	1.1128	0.066*	
C4	0.47982 (19)	0.18667 (18)	0.49997 (17)	0.0408 (4)	
C15	0.63517 (19)	0.1857 (2)	0.46986 (19)	0.0492 (5)	
H15A	0.6738	0.1108	0.5088	0.059*	
H15B	0.6298	0.1666	0.3723	0.059*	
C16	0.6984 (3)	0.4263 (2)	0.4602 (2)	0.0663 (6)	
H16A	0.7603	0.5112	0.5111	0.080*	
H16B	0.5954	0.4319	0.4656	0.080*	
C18	0.7341 (2)	0.4114 (2)	0.2047 (2)	0.0671 (6)	
H18	0.7476	0.4066	0.1147	0.081*	
C17	0.7173 (2)	0.4175 (2)	0.3175 (2)	0.0567 (5)	
O6	0.73584 (14)	0.31229 (15)	0.52126 (12)	0.0566 (4)	
O1	0.10633 (13)	0.11960 (14)	0.31046 (11)	0.0512 (4)	
O3	0.28037 (13)	0.24304 (13)	0.78669 (11)	0.0463 (3)	
O4	0.26536 (15)	0.40777 (14)	1.03810 (11)	0.0512 (3)	
O5	0.19356 (13)	0.66270 (14)	1.11802 (12)	0.0506 (3)	
O2	−0.08593 (13)	0.17487 (13)	0.08915 (11)	0.0490 (3)	

### Atomic displacement parameters (Å^2^)

**Table:**

	*U*^11^	*U*^22^	*U*^33^	*U*^12^	*U*^13^	*U*^23^
C1	0.0395 (9)	0.0443 (10)	0.0403 (10)	0.0095 (8)	0.0102 (7)	0.0095 (8)
C2	0.0433 (9)	0.0424 (10)	0.0335 (9)	0.0107 (8)	0.0051 (7)	0.0089 (7)
C3	0.0487 (10)	0.0493 (11)	0.0342 (9)	0.0136 (8)	0.0121 (8)	0.0088 (8)
C5	0.0413 (9)	0.0467 (11)	0.0391 (10)	0.0050 (8)	0.0047 (7)	0.0095 (8)
C6	0.0464 (10)	0.0416 (10)	0.0313 (9)	0.0067 (8)	0.0113 (7)	0.0076 (7)
C8	0.0416 (9)	0.0574 (12)	0.0425 (10)	0.0079 (9)	0.0061 (8)	0.0098 (9)
C7	0.0414 (10)	0.0587 (12)	0.0398 (10)	0.0125 (9)	0.0097 (8)	0.0122 (9)
C9	0.0546 (11)	0.0577 (12)	0.0383 (10)	0.0145 (9)	−0.0011 (8)	0.0002 (9)
C10	0.0512 (11)	0.0671 (13)	0.0349 (10)	0.0101 (10)	0.0029 (8)	0.0043 (9)
C11	0.0608 (12)	0.0579 (12)	0.0353 (10)	0.0090 (10)	0.0023 (8)	0.0089 (9)
C12	0.0623 (11)	0.0568 (12)	0.0271 (9)	0.0020 (9)	0.0092 (8)	0.0103 (8)
C13	0.0495 (10)	0.0712 (14)	0.0298 (9)	0.0012 (10)	0.0071 (8)	0.0074 (9)
C14	0.0589 (11)	0.0735 (14)	0.0369 (10)	0.0245 (11)	0.0067 (8)	0.0070 (9)
C4	0.0443 (10)	0.0416 (10)	0.0399 (10)	0.0101 (8)	0.0118 (8)	0.0106 (8)
C15	0.0441 (10)	0.0566 (12)	0.0510 (11)	0.0109 (9)	0.0140 (8)	0.0148 (9)
C16	0.0830 (15)	0.0566 (13)	0.0629 (14)	0.0066 (12)	0.0300 (12)	0.0094 (11)
C18	0.0694 (14)	0.0862 (17)	0.0565 (14)	0.0202 (12)	0.0215 (11)	0.0315 (12)
C17	0.0597 (12)	0.0608 (13)	0.0564 (13)	0.0135 (10)	0.0199 (10)	0.0201 (10)
O6	0.0530 (8)	0.0709 (10)	0.0438 (8)	−0.0001 (7)	0.0092 (6)	0.0150 (7)
O1	0.0435 (7)	0.0752 (9)	0.0350 (7)	0.0193 (6)	0.0034 (5)	0.0017 (6)
O3	0.0456 (7)	0.0607 (8)	0.0307 (6)	0.0048 (6)	0.0077 (5)	0.0051 (6)
O4	0.0700 (8)	0.0582 (8)	0.0279 (6)	0.0123 (7)	0.0118 (6)	0.0114 (6)
O5	0.0489 (7)	0.0630 (9)	0.0454 (7)	0.0154 (6)	0.0136 (6)	0.0163 (6)
O2	0.0529 (7)	0.0567 (8)	0.0336 (7)	0.0045 (6)	0.0002 (5)	0.0078 (6)

### Geometric parameters (Å, °)

**Table:**

C1—C2	1.376 (2)	C10—H10B	0.9700
C1—C6	1.398 (2)	C11—O4	1.409 (2)
C1—H1	0.9300	C11—H11A	0.9700
C2—O1	1.3682 (19)	C11—H11B	0.9700
C2—C3	1.396 (2)	C12—O4	1.423 (2)
C3—C4	1.373 (2)	C12—C13	1.482 (3)
C3—H3	0.9300	C12—H12A	0.9700
C5—C6	1.384 (2)	C12—H12B	0.9700
C5—C4	1.400 (2)	C13—O5	1.420 (2)
C5—H5	0.9300	C13—H13A	0.9700
C6—O3	1.3672 (19)	C13—H13B	0.9700
C8—O2	1.412 (2)	C14—O5	1.423 (2)
C8—C7	1.497 (2)	C14—C9^i^	1.492 (3)
C8—H8A	0.9700	C14—H14A	0.9700
C8—H8B	0.9700	C14—H14B	0.9700
C7—O1	1.428 (2)	C4—C15	1.510 (2)
C7—H7A	0.9700	C15—O6	1.418 (2)
C7—H7B	0.9700	C15—H15A	0.9700
C9—O2	1.418 (2)	C15—H15B	0.9700
C9—C14^i^	1.492 (3)	C16—O6	1.412 (3)
C9—H9A	0.9700	C16—C17	1.468 (3)
C9—H9B	0.9700	C16—H16A	0.9700
C10—O3	1.431 (2)	C16—H16B	0.9700
C10—C11	1.499 (2)	C18—C17	1.167 (3)
C10—H10A	0.9700	C18—H18	0.9300
			
C2—C1—C6	118.97 (15)	C10—C11—H11B	109.6
C2—C1—H1	120.5	H11A—C11—H11B	108.1
C6—C1—H1	120.5	O4—C12—C13	109.57 (14)
O1—C2—C1	125.21 (15)	O4—C12—H12A	109.8
O1—C2—C3	114.18 (14)	C13—C12—H12A	109.8
C1—C2—C3	120.60 (15)	O4—C12—H12B	109.8
C4—C3—C2	120.15 (15)	C13—C12—H12B	109.8
C4—C3—H3	119.9	H12A—C12—H12B	108.2
C2—C3—H3	119.9	O5—C13—C12	109.56 (15)
C6—C5—C4	119.21 (15)	O5—C13—H13A	109.8
C6—C5—H5	120.4	C12—C13—H13A	109.8
C4—C5—H5	120.4	O5—C13—H13B	109.8
O3—C6—C5	124.27 (15)	C12—C13—H13B	109.8
O3—C6—C1	114.76 (14)	H13A—C13—H13B	108.2
C5—C6—C1	120.95 (15)	O5—C14—C9^i^	109.25 (15)
O2—C8—C7	109.34 (15)	O5—C14—H14A	109.8
O2—C8—H8A	109.8	C9^i^—C14—H14A	109.8
C7—C8—H8A	109.8	O5—C14—H14B	109.8
O2—C8—H8B	109.8	C9^i^—C14—H14B	109.8
C7—C8—H8B	109.8	H14A—C14—H14B	108.3
H8A—C8—H8B	108.3	C3—C4—C5	120.11 (15)
O1—C7—C8	106.52 (14)	C3—C4—C15	119.87 (15)
O1—C7—H7A	110.4	C5—C4—C15	120.02 (16)
C8—C7—H7A	110.4	O6—C15—C4	113.54 (15)
O1—C7—H7B	110.4	O6—C15—H15A	108.9
C8—C7—H7B	110.4	C4—C15—H15A	108.9
H7A—C7—H7B	108.6	O6—C15—H15B	108.9
O2—C9—C14^i^	108.95 (16)	C4—C15—H15B	108.9
O2—C9—H9A	109.9	H15A—C15—H15B	107.7
C14^i^—C9—H9A	109.9	O6—C16—C17	113.58 (17)
O2—C9—H9B	109.9	O6—C16—H16A	108.8
C14^i^—C9—H9B	109.9	C17—C16—H16A	108.8
H9A—C9—H9B	108.3	O6—C16—H16B	108.8
O3—C10—C11	109.13 (15)	C17—C16—H16B	108.8
O3—C10—H10A	109.9	H16A—C16—H16B	107.7
C11—C10—H10A	109.9	C17—C18—H18	180.0
O3—C10—H10B	109.9	C18—C17—C16	179.1 (2)
C11—C10—H10B	109.9	C16—O6—C15	113.57 (15)
H10A—C10—H10B	108.3	C2—O1—C7	119.54 (13)
O4—C11—C10	110.25 (15)	C6—O3—C10	117.49 (13)
O4—C11—H11A	109.6	C11—O4—C12	112.09 (13)
C10—C11—H11A	109.6	C13—O5—C14	112.83 (14)
O4—C11—H11B	109.6	C8—O2—C9	112.47 (14)
			
C6—C1—C2—O1	177.94 (16)	C5—C4—C15—O6	−55.4 (2)
C6—C1—C2—C3	−1.3 (3)	O6—C16—C17—C18	−90 (17)
O1—C2—C3—C4	−178.87 (15)	C17—C16—O6—C15	−69.0 (2)
C1—C2—C3—C4	0.4 (3)	C4—C15—O6—C16	−64.9 (2)
C4—C5—C6—O3	177.64 (16)	C1—C2—O1—C7	13.2 (3)
C4—C5—C6—C1	−1.0 (3)	C3—C2—O1—C7	−167.59 (15)
C2—C1—C6—O3	−177.18 (15)	C8—C7—O1—C2	−178.61 (15)
C2—C1—C6—C5	1.5 (3)	C5—C6—O3—C10	4.2 (2)
O2—C8—C7—O1	−67.06 (18)	C1—C6—O3—C10	−177.17 (16)
O3—C10—C11—O4	−67.2 (2)	C11—C10—O3—C6	−176.06 (15)
O4—C12—C13—O5	−57.51 (19)	C10—C11—O4—C12	−167.39 (15)
C2—C3—C4—C5	0.2 (3)	C13—C12—O4—C11	−168.70 (16)
C2—C3—C4—C15	179.74 (16)	C12—C13—O5—C14	−169.02 (14)
C6—C5—C4—C3	0.1 (3)	C9^i^—C14—O5—C13	−163.09 (15)
C6—C5—C4—C15	−179.47 (16)	C7—C8—O2—C9	173.50 (14)
C3—C4—C15—O6	125.04 (18)	C14^i^—C9—O2—C8	173.71 (15)

Symmetry codes: (i) −*x*, −*y*+1, −*z*+1.

### Hydrogen-bond geometry (Å, °)

**Table:**

*D*—H···*A*	*D*—H	H···*A*	*D*···*A*	*D*—H···*A*
C13—H13B···O6^ii^	0.97	2.49	3.247 (2)	135.
C18—H18···O4^iii^	0.93	2.54	3.203 (2)	128.
C18—H18···O5^iii^	0.93	2.52	3.431 (3)	166.

Symmetry codes: (ii) −*x*+1, −*y*+1, −*z*+2; (iii) −*x*+1, −*y*+1, −*z*+1.
